# (+)-[1-(4-Methoxy­benz­yl)pyrrolidin-2-yl]diphenyl­methanol

**DOI:** 10.1107/S1600536809011817

**Published:** 2009-04-18

**Authors:** Jia Jin, Si-Jia Xue, Xue-Fei Li, Zhi-Kun Fang

**Affiliations:** a Department of Chemistry, College of Life and Environmental Science, Shanghai Normal University, Shanghai, 200234, People’s Republic of China

## Abstract

The title compound, C_25_H_27_NO_2_, was obtained as the product of a Grignard reagent and an inter­mediate ester synthesized from L-(-)-proline. The asymmetric unit contains two independent mol­ecules, both of which feature an intra­molecular O—H⋯N hydrogen bond. In one of the mol­ecules, the pyrrolidine ring is disordered over two orientations in a 0.63 (3):0.37 (3) ratio.

## Related literature

For the synthesis, see: Baker *et al.* (1991 ▶); Zhao *et al.* (1999 ▶). For background on the applications of this family of compounds, see: Kagabu *et al.* (2007 ▶).
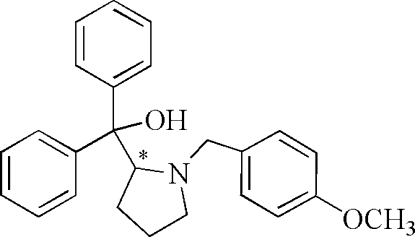


## Experimental

### 

#### Crystal data

C_25_H_27_NO_2_
*M* *_r_* = 373.48
Monoclinic, 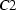

*a* = 33.131 (4) Å
*b* = 5.9916 (7) Å
*c* = 21.472 (3) Å
β = 97.715 (3)°
*V* = 4223.8 (9) Å^3^
*Z* = 8
Mo *K*α radiation
μ = 0.07 mm^−1^
*T* = 292 K
0.20 × 0.10 × 0.04 mm

#### Data collection

Bruker SMART CCD diffractometer
Absorption correction: none
20497 measured reflections
4093 independent reflections
2040 reflections with *I* > 2σ(*I*)
*R*_int_ = 0.107

#### Refinement

*R*[*F* ^2^ > 2σ(*F* ^2^)] = 0.067
*wR*(*F* ^2^) = 0.179
*S* = 0.93
4093 reflections
536 parameters
7 restraints
H-atom parameters constrained
Δρ_max_ = 0.19 e Å^−3^
Δρ_min_ = −0.16 e Å^−3^

### 

Data collection: *SMART* (Bruker, 2000 ▶); cell refinement: *SAINT* (Bruker, 2000 ▶); data reduction: *SAINT*; program(s) used to solve structure: *SHELXS97* (Sheldrick, 2008 ▶); program(s) used to refine structure: *SHELXL97* (Sheldrick, 2008 ▶); molecular graphics: *SHELXTL* (Sheldrick, 2008 ▶); software used to prepare material for publication: *SHELXTL*.

## Supplementary Material

Crystal structure: contains datablocks I, global. DOI: 10.1107/S1600536809011817/hb2936sup1.cif

Structure factors: contains datablocks I. DOI: 10.1107/S1600536809011817/hb2936Isup2.hkl

Additional supplementary materials:  crystallographic information; 3D view; checkCIF report

**Fig. 2.**
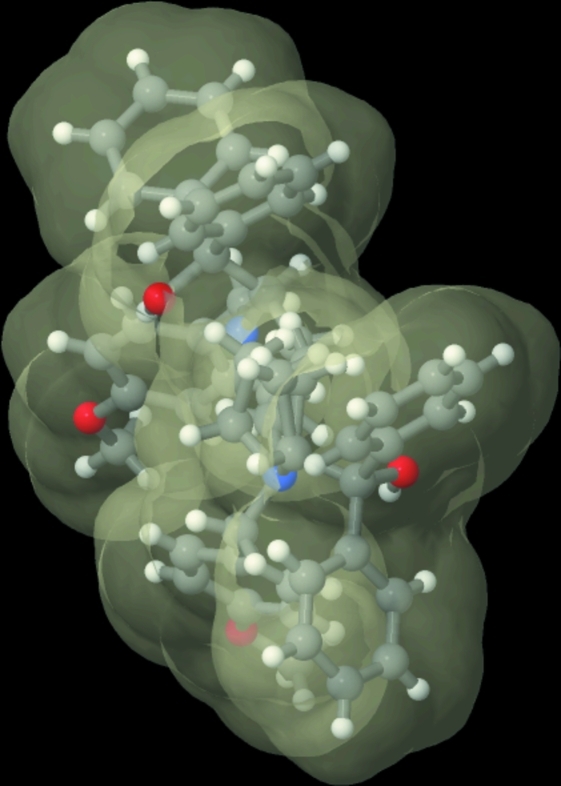
Supplementary figure.

Enhanced figure: interactive version of Fig. 2

## Figures and Tables

**Table 1:** Hydrogen-bond geometry (Å, °)

*D*—H⋯*A*	*D*—H	H⋯*A*	*D*⋯*A*	*D*—H⋯*A*
O1—H1⋯N1	0.82	2.32	2.673 (8)	106
O3—H3*A*⋯N2	0.82	2.33	2.672 (7)	106

**Fig. 1.**
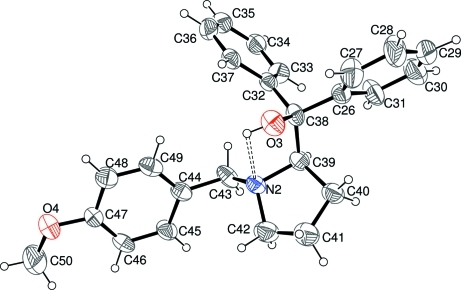
The molecular structure of the N2 molecule (I), showing 30% displacement ellipsoids for the non-hydrogen atoms. The hydrogen bond is shown as a double dashed line.

## supplementary crystallographic information

### Comment

Pyrrolidine derivatives have received much attention on their potential applications in chiral catalysis, medicine intermediate and pesticide (e.g. Kagabu *et al.*, 2007). We report here the synthesis and crystal structure of the title compound (I).

There are four aromatic rings in one molecule structure (Figure 1), namely a pyrrolidine ring (N2/C39—C42), phenyl rings (C26—C31), (C32—C37), (C44—C49) (Fig 1). the phenyl ring (C26—C31) and phenyl ring (C32—C37) forms dihedral angle of 71.5 (8)°; the phenyl ring (C32—C37) and phenyl ring (C44—C49) forms dihedral angle of 83.4 (6)°; the phenyl ring (C26—C31) and phenyl ring (C44—C49) forms dihedral angle of 41.1 (6)°. It is interesting that the dihedral angle of the phenyl ring (C26—C31) and phenyl ring (C44—C49) are smaller than the other two dihedral angles. It probably because that the —OCH_3_ effect the phenyl ring (C44—C49). Another structure feature that should be mentioned here is the N—C bond length data (C18—N1 1.45 Å and C43—N2 1.45 Å) in the crystal of compound (I) are between the standard C—N (1.47 Å) and C=N (1.26 Å), which shows that the nitrogen atom may have weak conjugation with the carbon atom (C18 and C43).

The title compound (I) belongs to monoclinic crystal system with space group C2. Each asymmetric unit contains two independent conformational isomer molecules, and two intramolecular hydrogen bonds (O3—H3A···N2) and (O1—H1···N1) are observed (Table 1) forming two five-membered rings (O1, H1, N1, C13, C14) and (O3, H3A, N2, C38, C39).

### Experimental

The title compound was prepared by the literature method (Zhao *et al.*, 1999; Baker *et al.*, 1991) and crystals were grown using volatilization of dichloromethane and methanol solution at room temperature. ^1^H NMR (DMSO-d_6_, 400 MHz): 6.67–7.66 (m, 14H, Ph—H), 4.95(s, 1H, —OH),3.95(m, 1H, pyrrol N—H), 3.61(s, 3H, —OCH_3_), 2.95(m, 2H, CH_2_), 2.69(m, 1H, pyrrol N—H), 2.17(m, 1H, pyrrol N—H), 1.78(m, 1H, pyrrol N—H), 1.45(m, 3H, pyrrol N—H); Analysis calculated for C_25_H_27_NO_2_: C 80.61, H 7.04, N 3.76%; found: C 80.40, H 7.29, N 3.75%. Colourless plates of (I) suitable for single-crystal X-ray diffraction were selected directly from the sample as prepared.

### Refinement

Anomalous dispersion was negligible and Friedel pairs were merged before refinement. The H atoms were placed in idealised locations (C—H = 0.93–0.98Å, O—H = 0.82Å) and refined as riding with U_iso_(H) = 1.2U_eq_(C) or 1.5U_eq_(methyl C, O).

### Crystal data

**Table:**

C_25_H_27_NO_2_	*F*(000) = 1600
*M**_r_* = 373.48	*D*_x_ = 1.175 Mg m^−^^3^
Monoclinic, *C*2	Melting point: 391 K
Hall symbol: C 2y	Mo *K*α radiation, λ = 0.71073 Å
*a* = 33.131 (4) Å	Cell parameters from 1185 reflections
*b* = 5.9916 (7) Å	θ = 2.5–17.0°
*c* = 21.472 (3) Å	µ = 0.07 mm^−^^1^
β = 97.715 (3)°	*T* = 292 K
*V* = 4223.8 (9) Å^3^	Plate, colourless
*Z* = 8	0.20 × 0.10 × 0.04 mm

### Data collection

**Table:**

Bruker SMART CCD diffractometer	2040 reflections with *I* > 2σ(*I*)
Radiation source: fine-focus sealed tube	*R*_int_ = 0.107
graphite	θ_max_ = 25.0°, θ_min_ = 1.7°
ω scans	*h* = −39→39
20497 measured reflections	*k* = −7→7
4093 independent reflections	*l* = −25→25

### Refinement

**Table:**

Refinement on *F*^2^	Secondary atom site location: difference Fourier map
Least-squares matrix: full	Hydrogen site location: inferred from neighbouring sites
*R*[*F*^2^ > 2σ(*F*^2^)] = 0.067	H-atom parameters constrained
*wR*(*F*^2^) = 0.179	*w* = 1/[σ^2^(*F*_o_^2^) + (0.0935*P*)^2^] where *P* = (*F*_o_^2^ + 2*F*_c_^2^)/3
*S* = 0.93	(Δ/σ)_max_ < 0.001
4093 reflections	Δρ_max_ = 0.19 e Å^−^^3^
536 parameters	Δρ_min_ = −0.16 e Å^−^^3^
7 restraints	Extinction correction: *SHELXL97* (Sheldrick, 2008), Fc^*^=kFc[1+0.001xFc^2^λ^3^/sin(2θ)]^-1/4^
Primary atom site location: structure-invariant direct methods	Extinction coefficient: 0.0016 (4)

### Special details

**Table:**

Geometry. All e.s.d.'s (except the e.s.d. in the dihedral angle between two l.s. planes) are estimated using the full covariance matrix. The cell e.s.d.'s are taken into account individually in the estimation of e.s.d.'s in distances, angles and torsion angles; correlations between e.s.d.'s in cell parameters are only used when they are defined by crystal symmetry. An approximate (isotropic) treatment of cell e.s.d.'s is used for estimating e.s.d.'s involving l.s. planes.
Refinement. Refinement of *F*^2^ against ALL reflections. The weighted *R*-factor *wR* and goodness of fit *S* are based on *F*^2^, conventional *R*-factors *R* are based on *F*, with *F* set to zero for negative *F*^2^. The threshold expression of *F*^2^ > σ(*F*^2^) is used only for calculating *R*-factors(gt) *etc*. and is not relevant to the choice of reflections for refinement. *R*-factors based on *F*^2^ are statistically about twice as large as those based on *F*, and *R*- factors based on ALL data will be even larger.

### Fractional atomic coordinates and isotropic or equivalent isotropic displacement parameters (Å^2^)

**Table:**

	*x*	*y*	*z*	*U*_iso_*/*U*_eq_	Occ. (<1)
C1	0.08941 (17)	0.3509 (11)	0.3865 (3)	0.0601 (17)	
C2	0.0964 (2)	0.1590 (16)	0.4202 (3)	0.099 (2)	
H2	0.1087	0.0383	0.4030	0.119*	
C3	0.0852 (3)	0.143 (2)	0.4800 (4)	0.122 (3)	
H3	0.0911	0.0115	0.5026	0.146*	
C4	0.0666 (3)	0.303 (2)	0.5063 (4)	0.112 (3)	
H4	0.0585	0.2841	0.5458	0.135*	
C5	0.0596 (3)	0.4983 (19)	0.4745 (3)	0.114 (3)	
H5	0.0472	0.6161	0.4928	0.136*	
C6	0.0709 (2)	0.5235 (14)	0.4141 (3)	0.097 (3)	
H6	0.0659	0.6577	0.3927	0.117*	
C7	0.04379 (19)	0.6188 (11)	0.2672 (3)	0.0637 (17)	
H7	0.0527	0.7343	0.2945	0.076*	
C8	0.0106 (2)	0.6517 (14)	0.2239 (3)	0.0721 (18)	
H8	−0.0030	0.7877	0.2226	0.087*	
C9	−0.0031 (2)	0.4887 (18)	0.1823 (3)	0.088 (2)	
H9	−0.0257	0.5126	0.1523	0.106*	
C10	0.0170 (2)	0.2886 (14)	0.1852 (4)	0.090 (2)	
H10	0.0079	0.1755	0.1571	0.109*	
C11	0.0506 (2)	0.2531 (12)	0.2294 (3)	0.0760 (19)	
H11	0.0640	0.1163	0.2305	0.091*	
C12	0.06482 (16)	0.4176 (10)	0.2720 (2)	0.0485 (15)	
C13	0.10208 (18)	0.3747 (12)	0.3204 (3)	0.0633 (17)	
C14	0.13520 (17)	0.5563 (14)	0.3190 (2)	0.073 (2)	
H14	0.1237	0.7068	0.3201	0.087*	
C17	0.2001 (3)	0.488 (5)	0.2787 (6)	0.099 (7)	0.63 (3)
H17A	0.2148	0.6269	0.2761	0.119*	0.63 (3)
H17B	0.2100	0.3793	0.2510	0.119*	0.63 (3)
C16	0.2048 (4)	0.401 (5)	0.3470 (5)	0.091 (7)	0.63 (3)
H16A	0.2019	0.2403	0.3482	0.109*	0.63 (3)
H16B	0.2310	0.4428	0.3698	0.109*	0.63 (3)
C15	0.1699 (8)	0.519 (5)	0.3738 (15)	0.077 (8)	0.63 (3)
H15A	0.1791	0.6605	0.3923	0.093*	0.63 (3)
H15B	0.1604	0.4271	0.4061	0.093*	0.63 (3)
C15'	0.1681 (16)	0.600 (12)	0.379 (3)	0.117 (17)	0.37 (3)
H15C	0.1632	0.7412	0.3986	0.140*	0.37 (3)
H15D	0.1681	0.4811	0.4093	0.140*	0.37 (3)
C16'	0.2083 (9)	0.605 (11)	0.3506 (11)	0.134 (16)	0.37 (3)
H16C	0.2252	0.7290	0.3676	0.161*	0.37 (3)
H16D	0.2233	0.4673	0.3597	0.161*	0.37 (3)
C17'	0.1961 (6)	0.633 (6)	0.2798 (10)	0.089 (10)	0.37 (3)
H17C	0.1942	0.7899	0.2690	0.107*	0.37 (3)
H17D	0.2163	0.5642	0.2572	0.107*	0.37 (3)
C18	0.14033 (19)	0.6485 (12)	0.2070 (3)	0.0732 (19)	
H18A	0.1475	0.8043	0.2138	0.088*	
H18B	0.1108	0.6383	0.2003	0.088*	
C19	0.15663 (18)	0.5684 (12)	0.1493 (3)	0.0616 (17)	
C20	0.18472 (19)	0.6911 (13)	0.1207 (3)	0.0732 (19)	
H20	0.1933	0.8296	0.1369	0.088*	
C21	0.2001 (2)	0.6082 (14)	0.0680 (3)	0.077 (2)	
H21	0.2196	0.6895	0.0505	0.092*	
C22	0.1868 (2)	0.4085 (14)	0.0416 (3)	0.0696 (19)	
C23	0.15938 (19)	0.2875 (12)	0.0687 (3)	0.0729 (18)	
H23	0.1500	0.1518	0.0514	0.087*	
C24	0.14537 (19)	0.3664 (13)	0.1223 (3)	0.0725 (19)	
H24	0.1274	0.2779	0.1412	0.087*	
C25	0.1898 (2)	0.1469 (16)	−0.0416 (3)	0.110 (3)	
H25A	0.1958	0.0253	−0.0128	0.165*	
H25B	0.2034	0.1238	−0.0778	0.165*	
H25C	0.1609	0.1544	−0.0545	0.165*	
C26	0.33222 (18)	0.4931 (11)	0.3688 (3)	0.0618 (16)	
C27	0.3166 (2)	0.6886 (14)	0.3858 (4)	0.097 (2)	
H27	0.3166	0.8109	0.3592	0.117*	
C28	0.3004 (3)	0.7125 (19)	0.4421 (5)	0.130 (4)	
H28	0.2915	0.8520	0.4534	0.156*	
C29	0.2977 (3)	0.545 (2)	0.4790 (4)	0.117 (3)	
H29	0.2848	0.5598	0.5146	0.140*	
C30	0.3142 (3)	0.343 (2)	0.4651 (4)	0.115 (3)	
H30	0.3142	0.2237	0.4928	0.138*	
C31	0.3311 (2)	0.3178 (14)	0.4090 (3)	0.093 (2)	
H31	0.3416	0.1803	0.3991	0.112*	
C32	0.39796 (18)	0.4804 (10)	0.3256 (2)	0.0550 (15)	
C33	0.4203 (2)	0.3095 (12)	0.3570 (3)	0.0680 (18)	
H33	0.4070	0.1805	0.3670	0.082*	
C34	0.4615 (2)	0.3257 (13)	0.3735 (3)	0.0731 (19)	
H34	0.4756	0.2096	0.3953	0.088*	
C35	0.4820 (2)	0.5112 (17)	0.3582 (3)	0.085 (2)	
H35	0.5101	0.5197	0.3689	0.102*	
C36	0.4613 (2)	0.6840 (14)	0.3273 (3)	0.079 (2)	
H36	0.4754	0.8106	0.3175	0.094*	
C37	0.4193 (2)	0.6723 (11)	0.3103 (3)	0.0661 (18)	
H37	0.4053	0.7902	0.2890	0.079*	
C38	0.35166 (18)	0.4709 (10)	0.3084 (3)	0.0587 (16)	
C39	0.33731 (17)	0.2594 (11)	0.2707 (2)	0.0595 (16)	
H39	0.3512	0.1278	0.2904	0.071*	
C40	0.29109 (17)	0.2231 (13)	0.2623 (3)	0.075 (2)	
H40A	0.2846	0.0794	0.2793	0.090*	
H40B	0.2779	0.3390	0.2839	0.090*	
C41	0.2770 (2)	0.232 (2)	0.1931 (3)	0.126 (4)	
H41A	0.2561	0.1207	0.1811	0.151*	
H41B	0.2661	0.3781	0.1808	0.151*	
C42	0.3128 (2)	0.1855 (15)	0.1642 (3)	0.093 (2)	
H42A	0.3104	0.2513	0.1226	0.111*	
H42B	0.3166	0.0258	0.1605	0.111*	
C43	0.38595 (19)	0.1928 (12)	0.1945 (2)	0.0728 (19)	
H43A	0.3843	0.0312	0.1946	0.087*	
H43B	0.4064	0.2372	0.2289	0.087*	
C44	0.39916 (18)	0.2683 (12)	0.1334 (3)	0.0614 (16)	
C45	0.3946 (2)	0.1367 (12)	0.0805 (3)	0.0738 (19)	
H45	0.3817	−0.0006	0.0819	0.089*	
C46	0.4086 (2)	0.2006 (15)	0.0252 (3)	0.087 (2)	
H46	0.4057	0.1063	−0.0095	0.105*	
C47	0.4267 (2)	0.4038 (14)	0.0225 (3)	0.075 (2)	
C48	0.4315 (2)	0.5416 (13)	0.0744 (3)	0.081 (2)	
H48	0.4440	0.6801	0.0732	0.097*	
C49	0.4172 (2)	0.4680 (13)	0.1279 (3)	0.075 (2)	
H49	0.4201	0.5619	0.1627	0.090*	
C50	0.4464 (3)	0.326 (2)	−0.0772 (4)	0.193 (6)	
H50A	0.4671	0.2198	−0.0620	0.289*	
H50B	0.4540	0.4015	−0.1132	0.289*	
H50C	0.4210	0.2487	−0.0888	0.289*	
N1	0.15568 (14)	0.5227 (11)	0.2625 (2)	0.0742 (16)	
N2	0.34699 (14)	0.2840 (9)	0.20507 (19)	0.0600 (13)	
O1	0.12066 (13)	0.1661 (8)	0.30748 (19)	0.0831 (14)	
H1	0.1278	0.1705	0.2724	0.125*	
O2	0.20340 (14)	0.3502 (10)	−0.0118 (2)	0.0925 (15)	
O3	0.33741 (13)	0.6575 (7)	0.26944 (19)	0.0773 (13)	
H3A	0.3482	0.6553	0.2372	0.116*	
O4	0.44186 (16)	0.4827 (12)	−0.0294 (2)	0.1126 (19)	

### Atomic displacement parameters (Å^2^)

**Table:**

	*U*^11^	*U*^22^	*U*^33^	*U*^12^	*U*^13^	*U*^23^
C1	0.062 (4)	0.069 (5)	0.048 (3)	0.006 (4)	0.001 (3)	0.000 (4)
C2	0.128 (7)	0.098 (6)	0.069 (5)	0.007 (5)	0.002 (4)	0.027 (5)
C3	0.164 (9)	0.119 (9)	0.081 (6)	0.005 (8)	0.014 (6)	0.035 (6)
C4	0.133 (8)	0.138 (9)	0.068 (5)	0.000 (7)	0.023 (5)	0.023 (7)
C5	0.143 (7)	0.139 (9)	0.063 (5)	0.032 (7)	0.032 (5)	0.004 (6)
C6	0.153 (7)	0.082 (5)	0.065 (4)	0.040 (6)	0.047 (5)	0.014 (4)
C7	0.076 (4)	0.053 (4)	0.063 (4)	0.019 (4)	0.015 (4)	−0.007 (3)
C8	0.066 (4)	0.080 (5)	0.067 (4)	0.009 (4)	−0.003 (4)	0.000 (4)
C9	0.065 (4)	0.125 (8)	0.072 (5)	0.003 (5)	−0.002 (4)	0.011 (5)
C10	0.098 (6)	0.070 (5)	0.095 (5)	−0.005 (5)	−0.019 (5)	−0.020 (5)
C11	0.088 (5)	0.064 (5)	0.074 (4)	0.007 (4)	0.005 (4)	−0.008 (4)
C12	0.051 (3)	0.051 (4)	0.045 (3)	−0.010 (3)	0.009 (3)	−0.005 (3)
C13	0.068 (4)	0.069 (5)	0.054 (4)	0.005 (4)	0.010 (3)	−0.008 (3)
C14	0.060 (4)	0.110 (6)	0.050 (4)	−0.009 (4)	0.010 (3)	−0.018 (4)
C17	0.062 (9)	0.15 (2)	0.083 (10)	0.032 (10)	0.009 (7)	0.011 (11)
C16	0.058 (8)	0.146 (18)	0.061 (8)	0.001 (10)	−0.022 (6)	−0.004 (9)
C15	0.054 (10)	0.13 (2)	0.041 (9)	−0.009 (10)	0.001 (7)	−0.019 (12)
C15'	0.09 (3)	0.14 (4)	0.11 (4)	0.00 (2)	−0.03 (2)	−0.01 (3)
C16'	0.09 (2)	0.19 (5)	0.12 (2)	−0.02 (2)	0.001 (16)	0.01 (2)
C17'	0.046 (14)	0.14 (3)	0.082 (17)	0.044 (15)	0.026 (11)	−0.009 (18)
C18	0.081 (4)	0.074 (5)	0.067 (4)	−0.001 (4)	0.021 (4)	0.005 (4)
C19	0.059 (4)	0.069 (5)	0.057 (4)	0.002 (4)	0.008 (3)	−0.001 (4)
C20	0.076 (4)	0.074 (5)	0.071 (4)	−0.010 (4)	0.014 (4)	−0.001 (4)
C21	0.077 (5)	0.089 (6)	0.069 (4)	−0.006 (4)	0.025 (4)	0.014 (4)
C22	0.069 (4)	0.092 (6)	0.049 (4)	0.008 (4)	0.013 (3)	0.002 (4)
C23	0.090 (5)	0.066 (4)	0.064 (4)	−0.005 (4)	0.017 (4)	0.006 (4)
C24	0.079 (5)	0.083 (6)	0.057 (4)	−0.009 (4)	0.018 (3)	0.012 (4)
C25	0.122 (7)	0.120 (8)	0.089 (5)	0.013 (6)	0.020 (5)	−0.020 (6)
C26	0.076 (4)	0.053 (4)	0.058 (4)	−0.004 (4)	0.015 (3)	−0.003 (4)
C27	0.115 (6)	0.072 (6)	0.114 (6)	0.006 (5)	0.049 (5)	0.001 (5)
C28	0.157 (9)	0.114 (8)	0.139 (8)	−0.020 (7)	0.096 (7)	−0.042 (7)
C29	0.118 (7)	0.146 (10)	0.098 (7)	−0.047 (7)	0.055 (5)	−0.042 (8)
C30	0.158 (8)	0.121 (8)	0.070 (5)	−0.024 (7)	0.033 (5)	0.002 (6)
C31	0.134 (6)	0.092 (6)	0.059 (4)	0.001 (5)	0.033 (4)	0.005 (5)
C32	0.072 (4)	0.048 (4)	0.046 (3)	0.000 (4)	0.014 (3)	−0.004 (3)
C33	0.072 (5)	0.063 (5)	0.068 (4)	0.007 (4)	0.004 (3)	0.004 (4)
C34	0.077 (5)	0.084 (6)	0.058 (4)	0.000 (4)	0.009 (4)	0.003 (4)
C35	0.075 (5)	0.110 (7)	0.073 (5)	−0.011 (6)	0.018 (4)	−0.019 (5)
C36	0.085 (5)	0.080 (6)	0.078 (5)	−0.017 (5)	0.037 (4)	−0.015 (5)
C37	0.101 (6)	0.050 (4)	0.051 (4)	−0.006 (4)	0.023 (3)	−0.005 (3)
C38	0.081 (4)	0.046 (4)	0.051 (3)	0.009 (3)	0.018 (3)	0.013 (3)
C39	0.065 (4)	0.061 (4)	0.051 (3)	0.006 (3)	0.003 (3)	0.006 (3)
C40	0.074 (4)	0.093 (6)	0.058 (4)	0.003 (4)	0.002 (3)	−0.003 (4)
C41	0.082 (5)	0.213 (12)	0.082 (5)	−0.020 (7)	0.011 (4)	−0.027 (7)
C42	0.107 (6)	0.097 (6)	0.068 (4)	−0.016 (5)	−0.011 (4)	0.007 (5)
C43	0.095 (5)	0.078 (5)	0.045 (3)	0.029 (4)	0.006 (3)	0.009 (4)
C44	0.068 (4)	0.062 (5)	0.052 (4)	0.014 (4)	0.000 (3)	−0.002 (4)
C45	0.102 (5)	0.064 (4)	0.058 (4)	−0.005 (4)	0.018 (4)	−0.014 (4)
C46	0.108 (6)	0.109 (7)	0.046 (4)	−0.015 (5)	0.013 (4)	−0.025 (4)
C47	0.078 (5)	0.095 (6)	0.051 (4)	−0.015 (4)	0.008 (4)	0.003 (4)
C48	0.092 (5)	0.076 (5)	0.076 (5)	−0.005 (4)	0.017 (4)	−0.012 (5)
C49	0.080 (5)	0.080 (6)	0.063 (4)	0.009 (4)	0.003 (4)	−0.018 (4)
C50	0.250 (13)	0.238 (15)	0.107 (7)	−0.108 (12)	0.088 (8)	−0.051 (9)
N1	0.055 (3)	0.121 (5)	0.045 (3)	0.016 (3)	0.002 (2)	−0.005 (3)
N2	0.063 (3)	0.069 (4)	0.046 (3)	0.005 (3)	0.001 (2)	0.002 (3)
O1	0.098 (3)	0.072 (3)	0.076 (3)	0.037 (3)	−0.001 (2)	−0.019 (3)
O2	0.097 (3)	0.114 (4)	0.072 (3)	−0.005 (3)	0.028 (3)	−0.009 (3)
O3	0.104 (3)	0.056 (3)	0.071 (3)	0.017 (3)	0.010 (2)	0.019 (3)
O4	0.128 (4)	0.146 (5)	0.068 (3)	−0.026 (4)	0.028 (3)	0.001 (4)

### Geometric parameters (Å, °)

**Table:**

C1—C2	1.362 (10)	C25—O2	1.421 (10)
C1—C6	1.376 (9)	C25—H25A	0.9600
C1—C13	1.540 (8)	C25—H25B	0.9600
C2—C3	1.388 (11)	C25—H25C	0.9600
C2—H2	0.9300	C26—C27	1.350 (9)
C3—C4	1.308 (12)	C26—C31	1.362 (9)
C3—H3	0.9300	C26—C38	1.529 (8)
C4—C5	1.359 (13)	C27—C28	1.394 (10)
C4—H4	0.9300	C27—H27	0.9300
C5—C6	1.404 (9)	C28—C29	1.291 (13)
C5—H5	0.9300	C28—H28	0.9300
C6—H6	0.9300	C29—C30	1.375 (14)
C7—C8	1.357 (8)	C29—H29	0.9300
C7—C12	1.389 (8)	C30—C31	1.403 (10)
C7—H7	0.9300	C30—H30	0.9300
C8—C9	1.360 (10)	C31—H31	0.9300
C8—H8	0.9300	C32—C33	1.384 (8)
C9—C10	1.369 (11)	C32—C37	1.411 (8)
C9—H9	0.9300	C32—C38	1.530 (8)
C10—C11	1.379 (9)	C33—C34	1.366 (8)
C10—H10	0.9300	C33—H33	0.9300
C11—C12	1.383 (8)	C34—C35	1.364 (11)
C11—H11	0.9300	C34—H34	0.9300
C12—C13	1.525 (8)	C35—C36	1.363 (10)
C13—O1	1.437 (8)	C35—H35	0.9300
C13—C14	1.548 (9)	C36—C37	1.394 (9)
C14—N1	1.482 (7)	C36—H36	0.9300
C14—C15	1.55 (4)	C37—H37	0.9300
C14—C15'	1.59 (8)	C38—O3	1.438 (7)
C14—H14	0.9800	C38—C39	1.545 (9)
C17—N1	1.481 (8)	C39—N2	1.493 (7)
C17—C16	1.542 (9)	C39—C40	1.533 (8)
C17—H17A	0.9700	C39—H39	0.9800
C17—H17B	0.9700	C40—C41	1.499 (8)
C16—C15	1.531 (10)	C40—H40A	0.9700
C16—H16A	0.9700	C40—H40B	0.9700
C16—H16B	0.9700	C41—C42	1.437 (9)
C15—H15A	0.9700	C41—H41A	0.9700
C15—H15B	0.9700	C41—H41B	0.9700
C15'—C16'	1.535 (11)	C42—N2	1.461 (8)
C15'—H15C	0.9700	C42—H42A	0.9700
C15'—H15D	0.9700	C42—H42B	0.9700
C16'—C17'	1.528 (10)	C43—N2	1.447 (7)
C16'—H16C	0.9700	C43—C44	1.507 (8)
C16'—H16D	0.9700	C43—H43A	0.9700
C17'—N1	1.493 (10)	C43—H43B	0.9700
C17'—H17C	0.9700	C44—C49	1.349 (9)
C17'—H17D	0.9700	C44—C45	1.375 (8)
C18—N1	1.444 (7)	C45—C46	1.384 (8)
C18—C19	1.496 (8)	C45—H45	0.9300
C18—H18A	0.9700	C46—C47	1.362 (10)
C18—H18B	0.9700	C46—H46	0.9300
C19—C24	1.371 (9)	C47—O4	1.367 (7)
C19—C20	1.392 (8)	C47—C48	1.378 (9)
C20—C21	1.392 (8)	C48—C49	1.374 (9)
C20—H20	0.9300	C48—H48	0.9300
C21—C22	1.372 (9)	C49—H49	0.9300
C21—H21	0.9300	C50—O4	1.415 (12)
C22—C23	1.353 (9)	C50—H50A	0.9600
C22—O2	1.381 (7)	C50—H50B	0.9600
C23—C24	1.381 (8)	C50—H50C	0.9600
C23—H23	0.9300	O1—H1	0.8200
C24—H24	0.9300	O3—H3A	0.8200
			
C2—C1—C6	117.3 (6)	H25A—C25—H25B	109.5
C2—C1—C13	121.3 (6)	O2—C25—H25C	109.5
C6—C1—C13	121.4 (6)	H25A—C25—H25C	109.5
C1—C2—C3	120.3 (9)	H25B—C25—H25C	109.5
C1—C2—H2	119.9	C27—C26—C31	117.0 (6)
C3—C2—H2	119.9	C27—C26—C38	121.7 (6)
C4—C3—C2	123.1 (9)	C31—C26—C38	121.3 (6)
C4—C3—H3	118.4	C26—C27—C28	122.0 (8)
C2—C3—H3	118.4	C26—C27—H27	119.0
C3—C4—C5	118.3 (8)	C28—C27—H27	119.0
C3—C4—H4	120.9	C29—C28—C27	121.2 (9)
C5—C4—H4	120.9	C29—C28—H28	119.4
C4—C5—C6	120.5 (9)	C27—C28—H28	119.4
C4—C5—H5	119.7	C28—C29—C30	119.3 (8)
C6—C5—H5	119.7	C28—C29—H29	120.4
C1—C6—C5	120.4 (8)	C30—C29—H29	120.4
C1—C6—H6	119.8	C29—C30—C31	119.8 (9)
C5—C6—H6	119.8	C29—C30—H30	120.1
C8—C7—C12	122.0 (6)	C31—C30—H30	120.1
C8—C7—H7	119.0	C26—C31—C30	120.6 (8)
C12—C7—H7	119.0	C26—C31—H31	119.7
C7—C8—C9	120.9 (7)	C30—C31—H31	119.7
C7—C8—H8	119.5	C33—C32—C37	117.7 (6)
C9—C8—H8	119.5	C33—C32—C38	122.9 (6)
C8—C9—C10	118.8 (7)	C37—C32—C38	119.4 (6)
C8—C9—H9	120.6	C34—C33—C32	121.6 (6)
C10—C9—H9	120.6	C34—C33—H33	119.2
C9—C10—C11	120.7 (7)	C32—C33—H33	119.2
C9—C10—H10	119.7	C35—C34—C33	120.5 (7)
C11—C10—H10	119.7	C35—C34—H34	119.8
C10—C11—C12	121.1 (7)	C33—C34—H34	119.8
C10—C11—H11	119.5	C36—C35—C34	120.2 (7)
C12—C11—H11	119.5	C36—C35—H35	119.9
C11—C12—C7	116.5 (5)	C34—C35—H35	119.9
C11—C12—C13	120.2 (6)	C35—C36—C37	120.5 (7)
C7—C12—C13	123.3 (5)	C35—C36—H36	119.7
O1—C13—C12	110.1 (5)	C37—C36—H36	119.7
O1—C13—C1	105.9 (5)	C36—C37—C32	119.6 (6)
C12—C13—C1	110.4 (4)	C36—C37—H37	120.2
O1—C13—C14	106.5 (5)	C32—C37—H37	120.2
C12—C13—C14	112.3 (5)	O3—C38—C26	106.5 (5)
C1—C13—C14	111.4 (5)	O3—C38—C32	110.6 (5)
N1—C14—C15	103.1 (7)	C26—C38—C32	108.3 (5)
N1—C14—C13	108.9 (5)	O3—C38—C39	106.2 (4)
C15—C14—C13	109.8 (11)	C26—C38—C39	112.4 (5)
N1—C14—C15'	110.1 (12)	C32—C38—C39	112.7 (5)
C15—C14—C15'	18 (3)	N2—C39—C40	104.0 (4)
C13—C14—C15'	121 (2)	N2—C39—C38	108.9 (5)
N1—C14—H14	111.5	C40—C39—C38	114.2 (5)
C15—C14—H14	111.5	N2—C39—H39	109.9
C13—C14—H14	111.5	C40—C39—H39	109.9
C15'—C14—H14	93.2	C38—C39—H39	109.9
N1—C17—C16	104.0 (8)	C41—C40—C39	106.5 (5)
N1—C17—H17A	110.9	C41—C40—H40A	110.4
C16—C17—H17A	110.9	C39—C40—H40A	110.4
N1—C17—H17B	110.9	C41—C40—H40B	110.4
C16—C17—H17B	110.9	C39—C40—H40B	110.4
H17A—C17—H17B	109.0	H40A—C40—H40B	108.6
C15—C16—C17	102.5 (15)	C42—C41—C40	104.8 (6)
C15—C16—H16A	111.3	C42—C41—H41A	110.8
C17—C16—H16A	111.3	C40—C41—H41A	110.8
C15—C16—H16B	111.3	C42—C41—H41B	110.8
C17—C16—H16B	111.3	C40—C41—H41B	110.8
H16A—C16—H16B	109.2	H41A—C41—H41B	108.9
C16—C15—C14	107.7 (19)	C41—C42—N2	106.4 (6)
C16—C15—H15A	110.2	C41—C42—H42A	110.5
C14—C15—H15A	110.2	N2—C42—H42A	110.5
C16—C15—H15B	110.2	C41—C42—H42B	110.5
C14—C15—H15B	110.2	N2—C42—H42B	110.5
H15A—C15—H15B	108.5	H42A—C42—H42B	108.6
C16'—C15'—C14	103 (4)	N2—C43—C44	113.1 (5)
C16'—C15'—H15C	111.2	N2—C43—H43A	109.0
C14—C15'—H15C	111.2	C44—C43—H43A	109.0
C16'—C15'—H15D	111.2	N2—C43—H43B	109.0
C14—C15'—H15D	111.2	C44—C43—H43B	109.0
H15C—C15'—H15D	109.1	H43A—C43—H43B	107.8
C17'—C16'—C15'	105 (3)	C49—C44—C45	115.9 (6)
C17'—C16'—H16C	110.7	C49—C44—C43	121.7 (6)
C15'—C16'—H16C	110.7	C45—C44—C43	122.4 (7)
C17'—C16'—H16D	110.7	C44—C45—C46	122.6 (7)
C15'—C16'—H16D	110.7	C44—C45—H45	118.7
H16C—C16'—H16D	108.8	C46—C45—H45	118.7
N1—C17'—C16'	108.1 (18)	C47—C46—C45	118.9 (6)
N1—C17'—H17C	110.1	C47—C46—H46	120.5
C16'—C17'—H17C	110.1	C45—C46—H46	120.5
N1—C17'—H17D	110.1	C46—C47—O4	123.8 (7)
C16'—C17'—H17D	110.1	C46—C47—C48	120.3 (6)
H17C—C17'—H17D	108.4	O4—C47—C48	115.9 (7)
N1—C18—C19	112.9 (5)	C49—C48—C47	117.9 (7)
N1—C18—H18A	109.0	C49—C48—H48	121.0
C19—C18—H18A	109.0	C47—C48—H48	121.0
N1—C18—H18B	109.0	C44—C49—C48	124.4 (6)
C19—C18—H18B	109.0	C44—C49—H49	117.8
H18A—C18—H18B	107.8	C48—C49—H49	117.8
C24—C19—C20	116.1 (6)	O4—C50—H50A	109.5
C24—C19—C18	121.6 (6)	O4—C50—H50B	109.5
C20—C19—C18	122.2 (7)	H50A—C50—H50B	109.5
C19—C20—C21	120.6 (7)	O4—C50—H50C	109.5
C19—C20—H20	119.7	H50A—C50—H50C	109.5
C21—C20—H20	119.7	H50B—C50—H50C	109.5
C22—C21—C20	121.0 (6)	C18—N1—C17	119.8 (8)
C22—C21—H21	119.5	C18—N1—C14	117.1 (5)
C20—C21—H21	119.5	C17—N1—C14	112.0 (7)
C23—C22—C21	119.1 (6)	C18—N1—C17'	100.4 (13)
C23—C22—O2	126.0 (7)	C17—N1—C17'	34.3 (9)
C21—C22—O2	115.0 (7)	C14—N1—C17'	103.2 (10)
C22—C23—C24	119.7 (7)	C43—N2—C42	113.0 (5)
C22—C23—H23	120.1	C43—N2—C39	114.8 (4)
C24—C23—H23	120.1	C42—N2—C39	106.2 (5)
C19—C24—C23	123.4 (6)	C13—O1—H1	109.5
C19—C24—H24	118.3	C22—O2—C25	116.9 (6)
C23—C24—H24	118.3	C38—O3—H3A	109.5
O2—C25—H25A	109.5	C47—O4—C50	116.6 (8)
O2—C25—H25B	109.5		
			
C6—C1—C2—C3	−0.3 (11)	C33—C34—C35—C36	1.2 (10)
C13—C1—C2—C3	179.1 (7)	C34—C35—C36—C37	−0.8 (10)
C1—C2—C3—C4	2.2 (15)	C35—C36—C37—C32	0.4 (9)
C2—C3—C4—C5	−2.9 (16)	C33—C32—C37—C36	−0.5 (8)
C3—C4—C5—C6	1.9 (15)	C38—C32—C37—C36	178.4 (5)
C2—C1—C6—C5	−0.6 (11)	C27—C26—C38—O3	−18.6 (8)
C13—C1—C6—C5	180.0 (7)	C31—C26—C38—O3	163.5 (6)
C4—C5—C6—C1	−0.2 (14)	C27—C26—C38—C32	100.4 (7)
C12—C7—C8—C9	−1.2 (10)	C31—C26—C38—C32	−77.5 (7)
C7—C8—C9—C10	0.7 (10)	C27—C26—C38—C39	−134.5 (7)
C8—C9—C10—C11	−0.2 (11)	C31—C26—C38—C39	47.7 (8)
C9—C10—C11—C12	0.3 (11)	C33—C32—C38—O3	−172.7 (5)
C10—C11—C12—C7	−0.8 (9)	C37—C32—C38—O3	8.5 (7)
C10—C11—C12—C13	−180.0 (6)	C33—C32—C38—C26	70.9 (7)
C8—C7—C12—C11	1.2 (8)	C37—C32—C38—C26	−107.9 (6)
C8—C7—C12—C13	−179.6 (5)	C33—C32—C38—C39	−54.1 (7)
C11—C12—C13—O1	8.0 (7)	C37—C32—C38—C39	127.1 (5)
C7—C12—C13—O1	−171.2 (5)	O3—C38—C39—N2	46.9 (6)
C11—C12—C13—C1	−108.6 (6)	C26—C38—C39—N2	162.9 (5)
C7—C12—C13—C1	72.3 (7)	C32—C38—C39—N2	−74.3 (5)
C11—C12—C13—C14	126.5 (6)	O3—C38—C39—C40	−68.8 (6)
C7—C12—C13—C14	−52.6 (7)	C26—C38—C39—C40	47.2 (6)
C2—C1—C13—O1	−0.2 (8)	C32—C38—C39—C40	170.0 (5)
C6—C1—C13—O1	179.2 (6)	N2—C39—C40—C41	−0.7 (8)
C2—C1—C13—C12	118.9 (7)	C38—C39—C40—C41	117.9 (7)
C6—C1—C13—C12	−61.7 (8)	C39—C40—C41—C42	21.2 (10)
C2—C1—C13—C14	−115.7 (7)	C40—C41—C42—N2	−34.2 (10)
C6—C1—C13—C14	63.7 (8)	N2—C43—C44—C49	81.8 (7)
O1—C13—C14—N1	46.6 (6)	N2—C43—C44—C45	−100.0 (7)
C12—C13—C14—N1	−74.1 (6)	C49—C44—C45—C46	1.7 (10)
C1—C13—C14—N1	161.6 (5)	C43—C44—C45—C46	−176.6 (6)
O1—C13—C14—C15	−65.7 (10)	C44—C45—C46—C47	−1.5 (11)
C12—C13—C14—C15	173.7 (9)	C45—C46—C47—O4	179.4 (6)
C1—C13—C14—C15	49.4 (10)	C45—C46—C47—C48	0.9 (11)
O1—C13—C14—C15'	−82 (2)	C46—C47—C48—C49	−0.6 (11)
C12—C13—C14—C15'	157 (2)	O4—C47—C48—C49	−179.2 (6)
C1—C13—C14—C15'	33 (2)	C45—C44—C49—C48	−1.3 (10)
N1—C17—C16—C15	−31.8 (18)	C43—C44—C49—C48	177.0 (6)
C17—C16—C15—C14	29.8 (18)	C47—C48—C49—C44	0.8 (11)
N1—C14—C15—C16	−16.0 (18)	C19—C18—N1—C17	51.2 (15)
C13—C14—C15—C16	99.9 (16)	C19—C18—N1—C14	−167.6 (6)
C15'—C14—C15—C16	−130 (7)	C19—C18—N1—C17'	81.6 (13)
N1—C14—C15'—C16'	4(5)	C16—C17—N1—C18	166.6 (10)
C15—C14—C15'—C16'	75 (5)	C16—C17—N1—C14	23.6 (17)
C13—C14—C15'—C16'	132 (3)	C16—C17—N1—C17'	105 (3)
C14—C15'—C16'—C17'	16 (5)	C15—C14—N1—C18	−149.1 (12)
C15'—C16'—C17'—N1	−31 (4)	C13—C14—N1—C18	94.3 (7)
N1—C18—C19—C24	69.7 (8)	C15'—C14—N1—C18	−131 (3)
N1—C18—C19—C20	−108.7 (7)	C15—C14—N1—C17	−5.0 (17)
C24—C19—C20—C21	−0.3 (9)	C13—C14—N1—C17	−121.6 (14)
C18—C19—C20—C21	178.2 (6)	C15'—C14—N1—C17	13 (3)
C19—C20—C21—C22	2.6 (10)	C15—C14—N1—C17'	−39.9 (18)
C20—C21—C22—C23	−2.4 (10)	C13—C14—N1—C17'	−156.5 (15)
C20—C21—C22—O2	177.7 (6)	C15'—C14—N1—C17'	−22 (3)
C21—C22—C23—C24	0.0 (10)	C16'—C17'—N1—C18	154 (2)
O2—C22—C23—C24	179.8 (6)	C16'—C17'—N1—C17	−77 (3)
C20—C19—C24—C23	−2.2 (10)	C16'—C17'—N1—C14	32 (2)
C18—C19—C24—C23	179.3 (6)	C44—C43—N2—C42	72.0 (7)
C22—C23—C24—C19	2.4 (10)	C44—C43—N2—C39	−166.0 (6)
C31—C26—C27—C28	0.8 (11)	C41—C42—N2—C43	161.0 (7)
C38—C26—C27—C28	−177.2 (7)	C41—C42—N2—C39	34.4 (8)
C26—C27—C28—C29	−3.8 (14)	C40—C39—N2—C43	−145.4 (6)
C27—C28—C29—C30	5.6 (15)	C38—C39—N2—C43	92.5 (6)
C28—C29—C30—C31	−4.6 (15)	C40—C39—N2—C42	−19.8 (7)
C27—C26—C31—C30	0.2 (11)	C38—C39—N2—C42	−141.9 (6)
C38—C26—C31—C30	178.1 (7)	C23—C22—O2—C25	1.6 (9)
C29—C30—C31—C26	1.7 (13)	C21—C22—O2—C25	−178.6 (6)
C37—C32—C33—C34	1.0 (8)	C46—C47—O4—C50	−13.6 (11)
C38—C32—C33—C34	−177.9 (6)	C48—C47—O4—C50	165.0 (8)
C32—C33—C34—C35	−1.4 (9)		

### Hydrogen-bond geometry (Å, °)

**Table:**

*D*—H···*A*	*D*—H	H···*A*	*D*···*A*	*D*—H···*A*
O1—H1···N1	0.82	2.32	2.673 (8)	106
O3—H3A···N2	0.82	2.33	2.672 (7)	106
